# *Mapk*/Erk activation in an animal model of social deficits shows a possible link to autism

**DOI:** 10.1186/2040-2392-5-57

**Published:** 2014-12-22

**Authors:** Alireza Faridar, Dorothy Jones-Davis, Eric Rider, Jiang Li, Ilan Gobius, Laura Morcom, Linda J Richards, Saunak Sen, Elliott H Sherr

**Affiliations:** Department of Neurology, University of California, 675 Nelson Rising Way, Suite 214B, San Francisco, CA 94158 USA; Queensland Brain Institute, The University of Queensland, Brisbane, 4072 Australia; School of Biomedical Sciences, The University of Queensland, Brisbane, Queensland 4072 Australia; Epidemiology and Biostatistics, University of California, 550 16th Street, San Francisco, CA 94158 USA; Methodist Neurological Institute, 6550 Fannin St, Houston Texas, 77030 USA

## Abstract

**Background:**

There is converging preclinical and clinical evidence to suggest that the extracellular signal-regulated kinase (ERK) signaling pathway may be dysregulated in autism spectrum disorders.

**Method:**

We evaluated *Mapk*/Erk1/2, cellular proliferation and apoptosis in BTBR mice, as a preclinical model of Autism. We had previously generated 410 F2 mice from the cross of BTBR with B6. At that time, six different social behaviors in all F2 mice were evaluated and scored. In this study, eight mice at each extreme of the social behavioral spectrum were selected and the expression and activity levels of *Mapk*/Erk in the prefrontal cortex and cerebellum of these mice were compared. Finally, we compared the *Mapk*/Erk signaling pathway in brain and lymphocytes of the same mice, testing for correlation in the degree of kinase activation across these separate tissues.

**Results:**

Levels of phosphorylated Erk (p-Erk) were significantly increased in the brains of BTBR versus control mice. We also observed a significant association between juvenile social behavior and phosphorylated mitogen-activated protein kinase kinase (p-Mek) and p-Erk levels in the prefrontal cortex but not in the cerebellum. In contrast, we did not find a significant association between social behavior and total protein levels of either Mek or Erk. We also tested whether steady-state levels of Erk activation in the cerebral cortex in individual animals correlated with levels of Erk activation in lymphocytes, finding a significant relationship for this signaling pathway.

**Conclusion:**

These observations suggest that dysregulation of the ERK signaling pathway may be an important mediator of social behavior, and that measuring activation of this pathway in peripheral lymphocytes may serve as a surrogate marker for central nervous system (CNS) ERK activity, and possibly autistic behavior.

**Electronic supplementary material:**

The online version of this article (doi:10.1186/2040-2392-5-57) contains supplementary material, which is available to authorized users.

## Background

Autism spectrum disorders (ASD) are defined by the disruption of language and social function and self-stimulatory or repetitive behaviors. The etiologic heterogeneity of ASD has complicated progress towards understanding the biological mechanisms of autism and developing targeted therapies. Recently, however, advances in autism genetics and progress in the study of animal models have provided evidence to suggest that some intracellular pathways are commonly affected in autistic patients, particularly the mitogen-activated protein kinase (*MAPK*)/extracellular signal-related kinase (ERK) pathway [1]. Genome-wide association studies [2] and genomic copy number variant (CNV) analyses have identified enrichment in gene-sets involved in RAS (Rat Sarcoma)/*MAPK* signaling and kinase activation in ASD individuals [3]. Interestingly, a 593-kb deletion in chromosome 16p11.2, one of the most common CNVs associated with autism, contains the *MAPK3* (ERK1) gene [4, 5]. In addition, a number of single-gene mutations implicated in syndromes such as “RASopathy” disorders [1], fragile X syndrome [6], and tuberous sclerosis [7, 8], are also associated with disruption of the RAS/*MAPK* signaling pathway and lead to pleiotropic neurocognitive impairments, including ASD [1]. Furthermore, in mice, inactivation of *Mapk*(Erk2) in the forebrain results in alterations in behavior that have similarities with those seen in autism [9]. These findings provide preliminary evidence that regulation of ERK signaling may be broadly altered in autism.

The RAS/*MAPK* signaling pathway mediates the transmission of signals from cell surface receptors to cytoplasmic and nuclear effectors. Diverse groups of molecular adaptors bind RAS and/or RAP1 (RAS-related protein 1) and initiate downstream signaling through ERK [1]. Depending on enzyme kinetics, and sub-cellular distribution of each component, this pathway will mediate diverse cellular functions including proliferation, migration, differentiation, and cell survival [10]. In the nervous system, this pathway is additionally involved in a diverse array of activity-dependent neuronal events, including synaptic plasticity, long-term potentiation or depression (LTP and LTD), and memory formation [9, 11]. For example, in the transgenic mouse model of tuberous sclerosis, dysregulated ERK leads to impaired LTD, which was shown to mediate social behavioral deficits [7].

Recently, the inbred mouse strain BTBR T + tf/J (BTBR) has been studied as a possible preclinical model of autism [12], and we have recently shown that these behaviors are quantitatively linked to genetic loci [13]. A total of six quantitative trait loci, meeting genome-wide significance for three autism relevant behaviors in BTBR, were identified on chromosomes 1, 3, 9, 10, 12, and X. Moreover, in a recently published biochemical evaluation of BTBR mice, the authors identified upregulation of the ERK signaling pathway in the newborn mice, suggesting, but not demonstrating directly, that this elevation in Erk activation was linked with the autistic behavior in BTBR [14]. In this current study we evaluated the Ras**/***Mapk* signaling pathway in BTBR mice, and tested whether there was a correlation between the degree of activation of the Ras/*Mapk* pathways and autism-relevant traits, by testing intercrossed mice that all share varying degrees of the BTBR genome, allowing a stratified comparison of Erk activation and behavior. We also assessed whether levels of Erk activation in the brain correlated with levels of Erk activation in lymphocytes from the same animal. Both these lines of investigation provide preliminary evidence for the role of ERK activation in models of ASD and the capacity to monitor this activation in peripheral tissue.

## Methods

### Mice

BTBR, CD1 and C57BL/6 J (B6) lines were sourced from the Jackson Laboratory (Bar Harbor, Maine, United States). Mice from the same strain were bred either on site at the University of California, San Francisco or at The University of Queensland, under ethics approval from the respective University Animal Ethics Committees. Mice were weaned at P (postnatal day) 20 to 23 and then group-housed by sex in standard mouse cages containing two to four mice, following standard protocol. The day of vaginal plug was designated as E (embryonic day) 0 and the day of delivery as P0. BTBR and B6 were also bred and a total of 410 F2 mice were generated for behavioral testing over a period of two years in the laboratory of Dr Jacqueline Crawley at the NIMH (National Institute of Mental Health) in Bethesda, Maryland. The first cohort of 204 mice was generated by crossing F1 males and females derived from BTBR female and B6 male matings. The second cohort of 206 mice was generated by the reciprocal cross of F1 males and females derived from B6 female and BTBR male matings. This ensured equal representation of the BTBR and B6 X chromosomes in the final F2 cohort. Further details have been described previously [13].

### Autism-relevant behaviors evaluated in F2 mice

Juvenile reciprocal social interactions were assessed on P21. The test mouse and age- and sex-matched B6 control mice were simultaneously placed in the field and their interactions were videotaped for 10 minutes. Social behaviors including nose-to-nose sniff (sniffing the nose and snout region of the partner), front approach (moving towards the partner, in a head-on manner), and push-crawl (pushing the head underneath the partner’s body or squeezing between the wall or floor and the partner, and crawling over or under the partner’s body, combined as a single parameter) were evaluated and scored by a highly trained observer, using the Noldus Observer 5.0 software (Noldus Information Technology Wageningen, Netherlands).

Social approach was assayed in automated three-chambered apparatus between eight and 12 weeks of age. The test mouse was briefly confined to the center chamber while the clean novel object was placed in one of the side chambers. A novel mouse, previously habituated to the enclosure, was placed in an identical wire cup located in the other side chamber. The side containing the novel object and the novel mouse alternated between the left and right chambers across subjects. After both stimuli were positioned, the two side doors were simultaneously lifted and the subject was allowed access to all three chambers for 10 minutes. Number of entries and time spent in each of the three chambers were automatically detected by photocells embedded in the doorways and tallied by the software. In addition, time spent sniffing the novel mouse was scored by human observers [13].

### Protein extraction from mouse tissues and lymphocytes

P0 and P30 BTBR and B6 mice were euthanized by administration of inhaled CO_2_ followed by cervical dislocation. Whole brains were extracted and homogenized in ice-cold NP-40 lysis buffer containing 50 mM Tris (pH 7.5), 150 mM NaCl, 20 mM MgCl_2_, and 0.5% NP-40 with the addition of protein phosphatase inhibitor (PhosSTOP, Product number: 04906845001, Roche Diagnostics, Indianapolis, USA) and protease inhibitor cocktails (Complete Protease Inhibitor, Product number: 11697498001, Roche Diagnostics, Indianapolis, United states). After centrifugation (13,000 x-g, 15 minutes, 4°C), the supernatants were collected. The protein concentration was measured using the Bio-Rad protein assay (Life Science, Hercules, CA, USA) and all protein extracts were stored at −80°C.

Three-month-old F2 offspring from a cross of BTBR and B6 mice were anesthetized with ketamine and xylazine then perfused with phosphate buffered saline (PBS) and fixed with 4% paraformaldehyde via cardiac puncture, and the brains were extracted. One hemisphere was used for anatomic evaluation and proteins were isolated from the prefrontal cortex and cerebellum of the other hemisphere using the Qproteome FFPE kit (Qiagen, Hilden, Germany) [15].

To extract protein from lymphocytes, the spleens of the mice were removed and mechanically meshed through a cell strainer. Cells were washed with PBS and then 2 to 3 μl of red blood cell (RBC) lysis buffer (Biolegend, San Diego, USA) was added to the cell aliquot for 2 minutes to lyse the RBCs, and the remaining lymphocytes were pelleted and washed. Subsequently, NP-40 lysis buffer was added to the cell aliquot and the cellular extract subjected to centrifugation. The extracted protein supernatant was stored at −80°C.

### Protein electrophoresis and Western blot analysis

Samples were denatured in sample buffer by heating at 95°C for 5 minutes. A total of 20 micrograms of protein per lane was run on a 12% acrylamide gel for 2 hours at 100 V. The proteins were transferred to a PVDF membrane at 50 V for 1 hour and the PVDF membrane **(**Bio-RAD, Life Science Research, Hercules, USA) was then blocked for 1 hour with 5% dry milk in PBS with 0.1% Tween 20. Thereafter, the primary antibodies were applied overnight with continuous shaking at 4°C. The primary antibodies included rabbit mAb Ras, Phospho-mitogen-activated protein kinase kinase (MEK)1/2 (Ser217/221), rabbit mAb MEK1/2 (47E6), rabbit mAb Phospho-ERK (Thr 202/Tyr 204), p44/42 *MAPK*(ERK1/2), rabbit mAb β-actin (Cell Signaling). The blots were then washed and incubated with secondary antibody, HRP (Horseradish peroxidase) -linked anti-rabbit IgG, (Cell Signaling) for 1 hour at room temperature. Immunoreactive bands were visualized by using the enhanced chemiluminescence detection system (Pierce) and exposed to autoradiography film (HyBlot film CL). Densitometry was performed using ImageJ software, based on the standard protocol described via the National Institutes of Health (Bethesda, Maryland, United States). The intensity of the each protein is normalized to the actin signal obtained after stripping the same membrane and reprobing for actin in the same lane.

### Immunohistochemical analysis of cellular proliferation

Pregnant BTBR and control CD1 dams (n ≥6) were given an intraperitoneal injection of 5-Ethynyl-2′-deoxyuridine (EdU, 10 μg per kg of body weight, Invitrogen, Life Technologies, Grand Island, NY, USA) 30 minutes prior to sacrifice. Dams were then anesthetized with ketamine and xylazine and embryos collected at E14 and E17. E14 heads were immersion fixed in 4% paraformaldehyde (4% PFA) w/v in PBS pH7.4 (ProSciTech, Kirwan Australia), whilst E17 embryos were transcardially perfused with saline (0.9% NaCl w/v in H2O) followed by 4% PFA. Brains were extracted and sectioned at 50 μm thickness on the coronal orientation using a vibratome. Representative sections of the rostral telencephalon were mounted, post-fixed with 4% PFA for 20 minutes, and subjected to sodium citrate antigen retrieval (125°C at 15 psi for 4 minutes in sodium citrate buffer, 10 mM C6H5Na3O7, 2H2O, and 0.05% v/v Tween 20 in MilliQTM H2O, at pH 6.0). Sections were blocked in normal goat serum (10%)/0.2% Triton-X (Sigma-Aldrich, St Louis, Missouri, United States) in PBS for 30 minutes. EdU detection was performed using a Click-IT EdU Alexa Fluor 488 Imaging Kit (Invitrogen**,** Life Technologies, Grand Island, NY, USA), according to the manufacturer’s instructions. The slides were then light protected and re-blocked in 10% normal goat serum/0.2% Triton-X 100 in PBS for 2 hours. The following primary antibodies were applied to the slides in the blocking solution and left overnight: rabbit anti-phospho-histone H3 (Ser 10, 1:500, EMD Millipore, Darmstadt, Germany), rabbit anti-Pax6 (1:500, EMD Millipore, Darmstadt, Germany), rabbit anti-TBR2 (1:500, Abcam, Cambridge, USA) mouse anti-human Ki67 (1:500, BD Biosciences, San Jose, USA). After PBS washes, sections were then incubated for 3 hours with Alexa Fluor 555 donkey anti-rabbit IgG (1:500, Invitrogen, Life Technologies, Grand Island, NY, USA)) secondary antibody. Biotinylated donkey anti-mouse IgG (1:500, Jackson ImmunoResearch, West Grove, USA) was then applied for 1 hour. Following further PBS washes, Alexa Fluor 647 Streptavidin conjugate (1:500, Invitrogen Life Technologies, Grand Island, NY, USA)) was applied for one hour. Slides were counter-stained with nuclear marker DAPI (diamidino-2-phenylindole) (1:1000, Invitrogen, Life Technologies, Grand Island, NY, USA), washed and cover-slipped with ProLong Gold (Invitrogen Life Technologies, Grand Island, NY, USA). Images were obtained at 20x magnification in a single representative z-plane using an inverted spinning disk confocal microscope equipped with Hamamatsu Flash4.0 scientific CMOS camera (Hamamatsu, Japan) and acquired with Slidebook (3i Intelligent Imaging Innovations, Denver, USA). Images were pseudo-colored for presentation in Adobe Photoshop (Adobe Systems Incorporated, San Jose, United States).

### Quantification of cellular proliferation

Cell counts were performed on a region of interest from the neocortex, midline, and ganglionic eminence automatically using the spot analysis module in Imaris (Bitplane**,** Zurich, Switzerland). An appropriate threshold was set for detection of positive cells for each marker but kept constant between control and BTBR groups. Absolute cell counts were imported into Prism v.6 (GraphPad, La Jolla, USA) and presented as mean ± standard error. Statistical significance was determined at *P* <0.05, using the Mann-Whitney U test.

### Statistical analysis

All data are shown as mean ± SEM. Group comparisons are analyzed using the non- parametric Mann-Whitney U test, using GraphPad Prism v.6 (*P* <0.05 was considered statistically significant). Non-parametric correlation (Spearman correlation) was also conducted to compare paired measurements of *Mapk*/Erk levels in the brain and lymphocytes.

## Results

### Study design

**Figure 1:**
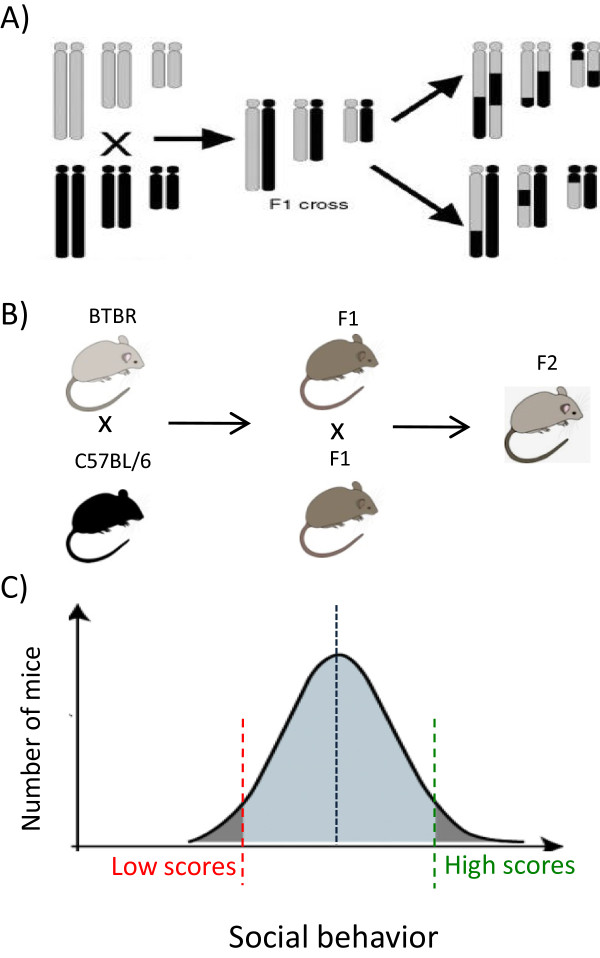
**Study design**. **A** and **B)** Intercrosses of BTBR and C57BL/6 mice were performed to produce F2 mice that have a mixed genetic background. **C)** Each juvenile and adult social behavior was evaluated and quantified in all 410 F2 mice. Mice with the lowest and highest scores of each social behavior were selected and *Mapk*/Erk activity in the prefrontal cortex and cerebellum were evaluated.

We evaluated Erk1/2 protein levels, cellular proliferation, and cellular apoptosis in the brains of BTBR mice, comparing these to wild-type mice as a control group. As part of a separate experiment, we previously generated 410 F2 mice from the crossing of BTBR mice with B6. We evaluated and scored six different juvenile and adult social behaviors in all F2 mice [13]. A total of eight mice at each extreme of the social behavior spectrum were selected and the expression, and activity levels of *Mapk*/Erk in the prefrontal cortex and cerebellum of these mice were compared (Figure 1). Finally, we compared the *Mapk*/Erk signaling pathway in the brains and lymphocytes of the same mice to evaluate the possibility of using lymphocytes as an indirect surrogate for Erk activation in the brain.

### The ERK signaling pathway is upregulated in BTBR mice

**Figure 2:**
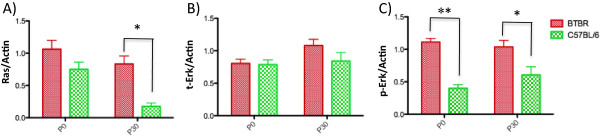
**Increased activity levels of** ***Mapk*** **/Erk signaling pathway in BTBR mice.** Western blot analysis of eight BTBR versus eight C57BL/6 mice. Brain lysates were evaluated at P0 and P30 time points with antibodies against Ras, t-Erk and p-Erk. Quantitative fold-changes of bands were assessed, after being normalized to actin levels **(A-** **C)**. Despite t-Erk levels being unchanged, the mean expression levels of Ras and p-Erk were significantly increased in BTBR mice at both P0 and P30 using the non-parametric Mann-Whitney U test (*P <0.05, **P <0.005, Bars represent the mean values, whiskers shows standard deviations).

To evaluate the Erk1/2 signaling pathway, we focused on Ras, Mek1/2 and Erk1/2 proteins and assessed the abundance of these and their phosphorylated isoforms in the brains of BTBR and B6 mice using Western blot analysis (Figure 2). In P0 mice, the relative expression level of RAS was 0.41 fold higher in BTBR mice compared to control B6 mice (trend level: *P* = 0.093). There was no difference in the total protein level of Erk (t-Erk) between the two strains (*P* = 0.930). We also evaluated the activity level of Erk1/2 by assessing the degree of phosphorylation of the Thr 202/Tyr 204 amino acids in the activation domain in Erk1 and the equivalent residues (Thr 185/Tyr 187) in Erk2. Despite the similarity in total protein levels for these kinases in BTBR and B6 mice, the relative amount of phosphorylated extracellular signal-regulated kinase (p-Erk)/actin in neonatal BTBR mice was 2.78 fold higher than in B6 mice (*P* = 0.002). We also evaluated the levels of p-ERK1 and p-ERK2 separately in BTBR and B6 mice, and we found that p-Erk was still elevated in BTBR compared to B6 mice (p-ERK1: 1.9 fold higher in BTBR, *P* = 0.004, p-Erk2: similarly 1.8 fold higher in BTBR, *P* <0.001). We also evaluated the kinase Mek, which is upstream of Erk. No significant difference was detected between the expression levels of p-MEK or total MEK levels in these two strains (*P* ≥0.05) (Western blot lanes of these experiments are shown in Additional file 1).

Next, we evaluated mice at P30 to determine whether this signaling pathway shows increased activation temporarily in the newborn period or whether this persists through the juvenile phase. Ras/actin and p-Erk/actin levels were significantly increased in P30 BTBR mice compared to B6 mice (RAS: 3.7 fold higher in BTBR, *P* = 0.007; p-Erk: 0.71 fold higher in BTBR, *P* = 0.026), whereas relative t-Erk levels remained unchanged (*P* = 0.179). To take into consideration the effect of total abundance of ERK in analyzing p-ERK levels, the ratio of (p-Erk/actin)/(t-Erk/actin) was also evaluated in P0 and P30 mice. In P0 animals, the ratio (p-Erk/actin)/(t-Erk/actin) was 1.59 fold higher in BTBR mice than in B6 (*P* = 0.005). However, this ratio was no longer significant at P30 (*P* = 0.281). In summary, these findings demonstrate that activation of the Erk pathway is elevated in the brains of BTBR mice, particularly in the newborn period, even though there may be experience-dependent or other developmental changes in behavior that correlate with changes in Erk activation in juvenile and adult mice [16, 17].

### Decreased cellular proliferation in embryonic BTBR mice

**Figure 3:**
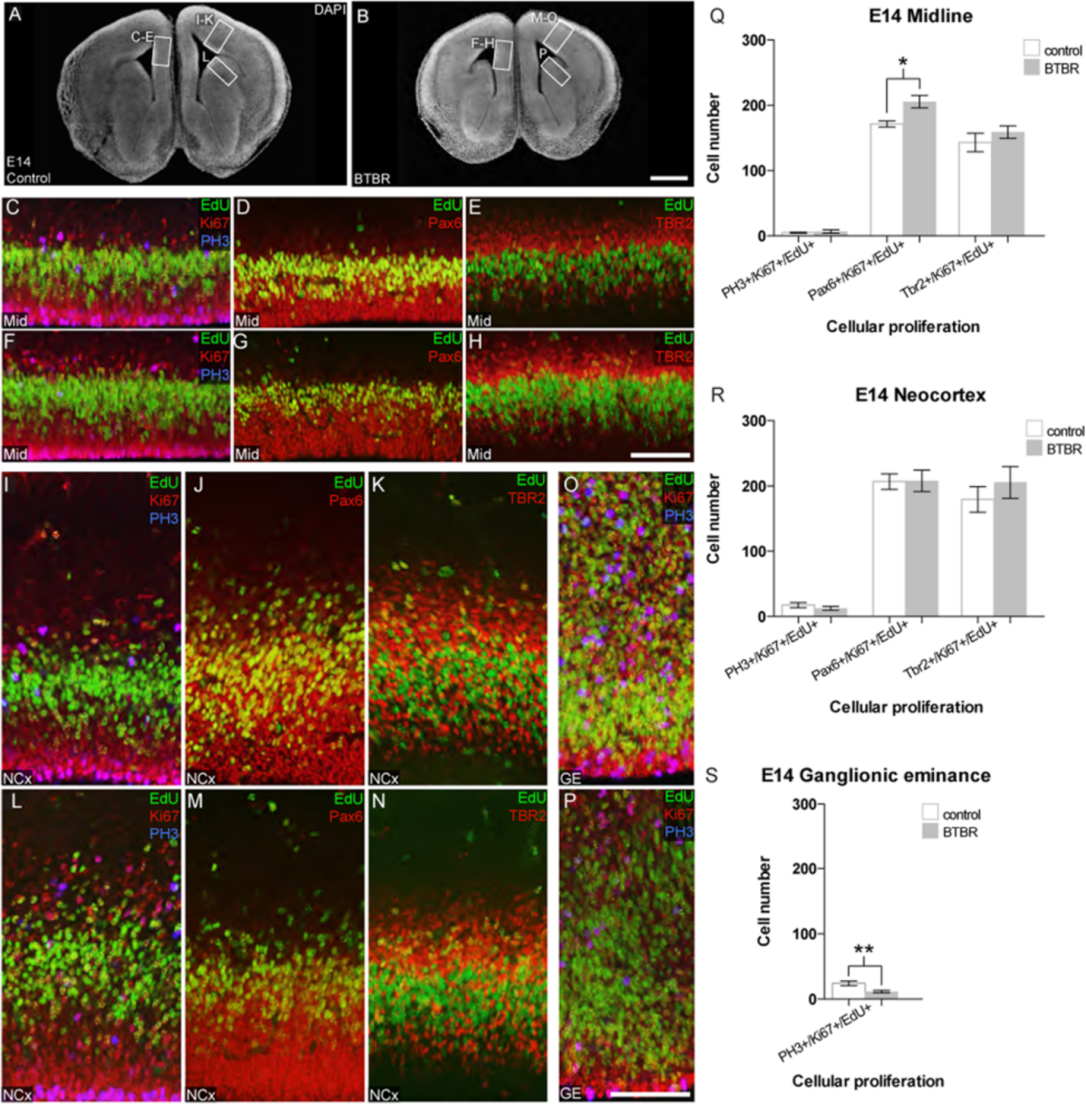
**BTBR mice exhibit altered cellular proliferation in the midline and ganglionic eminence at E14.** E14 control **(A)** and BTBR **(B)** mice (n ≥6) were injected with EdU 30 minutes prior to sacrifice and immunolabeled for nuclear marker DAPI (white), EdU (green), phospho-histone H3 (PH3, blue), and Ki67, Pax6, or Tbr2 (red). Cell counts were performed for single, double, and triple-labelled cells, where data for all counts are shown in Additional file 2: Figure S2. Representative regions from the midline of control **(C**-**E)** and BTBR **(F**-**H)** mice demonstrate that BTBR mice have increased incorporation of EdU, particularly into Pax6-positive cells, as quantified in **(Q)**. No difference was evident between the neocortex of control **(I**-**K)** and BTBR mice (**L**-**N**, and quantified in **R**). However, the ganglionic eminence of BTBR mice **(P)** exhibited decreased incorporation of EdU compared to control **(O)**, and reduced mitosis as indicated by PH3 staining (quantified in **S**). Scale bar in B represents 500 μm for A-B. Scale bar in H and P represents 100 μm for C-H and I-P respectively. Mann-Whitney U test for significance: *P <0.05, **P <0.01. Data are presented as mean ± SEM. (E14: Embryonic day 14; EDU, 5-Ethynyl-2′-deoxyuridine; DAPI, diamidino-2-phenylindole; Pax6: Paired Box 6; Tbr2,T-Box Brain Protein 2).

**Figure 4:**
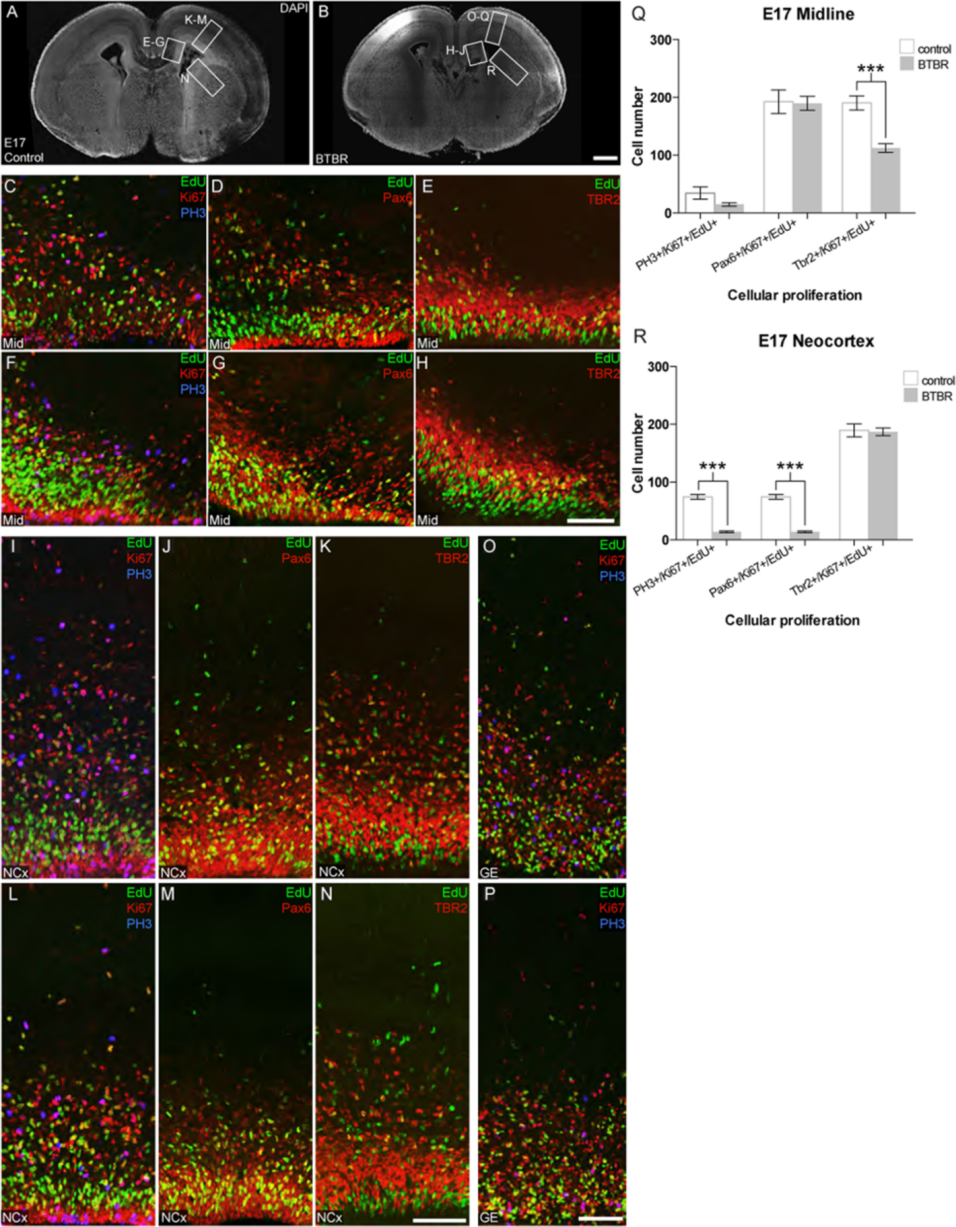
**BTBR mice exhibit reduced cellular proliferation in the midline and neocortex at E17 corresponding to a reduction in progenitor cells.** E17 control **(A)** and BTBR **(B)** mice (n ≥6) were injected with EdU 30 minutes prior to sacrifice and immunolabeled for nuclear marker DAPI (white), EdU (green), phospho-histone H3 (PH3, blue), and Ki67, Pax6, or Tbr2 (red). Cell counts were performed for single, double, and triple-labelled cells, where data for all counts are shown in Additional file 2: Figure S2. Representative regions from the midline of control **(C**-**E)** and BTBR **(F**-**H)** mice demonstrate that BTBR mice have reduced incorporation of EdU into Tbr2-positive cells, as quantified in **(Q)**. Furthermore, BTBR mice show reduced incorporation of EdU into mitotic cells (PH3-positive), and radial glia (Pax6-positive) in the neocortex **(L-N)** as compared to control animals (**I**-**K**, quantified in **R**). No differences in cellular proliferation were observed in the ganglionic eminence between control **(O)**, and BTBR mice **(P)**. Scale bar in B represents 500 μm for A-B. Scale bar in J represents 100 μm for E-J. Scale bar in Q represents 100 μm for K-M, O-Q. Scale bar in R represents 100 μm for N and R. Mann-Whitney U test for significance: ***P <0.001. Data are presented as mean ± SEM. (E17: Embryonic day 17; EDU, 5-Ethynyl-2′-deoxyuridine; DAPI, diamidino-2-phenylindole; Pax6, Paired Box 6; Tbr2,T-Box Brain Protein 2).

Dysregulation of the *MAPK*/ERK pathway has been implicated in altered neurogenesis or increased apoptosis, depending on the cell type and condition [17–20]. Because we observed an increase in the Erk signaling pathway, we wondered whether there might also be differences in neurogenesis and/or apoptosis in these mice. We therefore initially assessed neural cell proliferation in the forebrain at both E14 and E17 in BTBR (n ≥6 per age) and control mice (n ≥6 per age). To evaluate proliferation and any changes in cell cycle exit, immunohistochemistry with antibodies targeted to the mitotic marker phospho-histone H3 (PH3), the cortical radial glial marker Pax6, and the cortical intermediate progenitor marker Tbr2 were used in combination with the proliferation marker Ki67 and a 30 minute pulse of the thymidine analogue EdU to label proliferating cells entering S-phase (Figures 3 and 4). Representative regions of the cortical midline, neocortex, and ganglionic eminence (GE) were quantified in both BTBR and control mice. This analysis showed no differences in cell cycle exit (EdU-positive/Ki67-negative cells) between BTBR and control mice at either age (Additional file 2: Figure S2), however regional and age specific changes in proliferation were evident.

Within the cortical midline, we observed a significant increase (1.19 fold-change, *P* = 0.0122) in the number of radial glial cells entering S-phase at E14 (Pax6-positive/EdU-positive/Ki67-positive cells; Figure 3Q). Interestingly, this increase was not sustained at E17 (Figure 4Q). In contrast, we observed a decrease (0.59 fold-change, *P* = 0.0006) in the number of proliferating intermediate progenitor cells within the E17 cortical midline (Tbr2-positive/EdU-positive/Ki67-positive cells; Figure 4Q). This difference is specifically due to a decrease in the number of Tbr2-positive cells (0.79 fold-change, *P* = 0.0175) rather than a decrease in the total number of EdU-positive cells (Additional file 2: Figure S2D), suggesting that prolonged retention of radial glial progenitors at E14 may lead to a subsequent decrease in the number of intermediate progenitors at later stages. Within the neocortex, no significant changes in proliferation were evident at E14 (Figure 3R), however by E17, a marked reduction in mitotic cells (PH3-positive/EdU-positive/Ki67-positive: 0.18 fold-change, *P* = 0.0006) and proliferating radial glia (Pax6-positive/EdU-positive/Ki67-positive cells: 0.90 fold-change, *P* = 0.0006) was present (Figure 4R). Within the GE, we also observed a significant decrease (0.84 fold-change, *P* = 0.0087) in the number of mitotic cells throughout the GE at E14 (PH3-positive/EdU-positive/Ki67-positive cells, Figure 3S), however this difference was not sustained at E17 (Figure 4O, P; Additional file 2: Figure S2F). Note that the GE does not express high levels of Pax6 or Tbr2, compared to cortical structures and thus these markers cannot be used to define subpopulations of the GE (Additional file 3: Figure S3). Taken together, these results suggest that the genetic background of BTBR mice affects neural cell proliferation differentially throughout forebrain development, predominantly resulting in decreased proliferation relative to controls.

To determine whether apoptosis was affected in a similar manner, we then evaluated the apoptosis rate in the same regions. Immunohistochemical staining with the apoptotic marker cleaved caspase-3 showed no difference in cell death rates between BTBR and control mice (data not shown). These results suggest that there are developmental reductions in neural proliferation but no change in apoptosis in BTBR mice.

### Association between activity levels of *Mapk*/Erk and impairment in social behaviors

**Figure 5:**
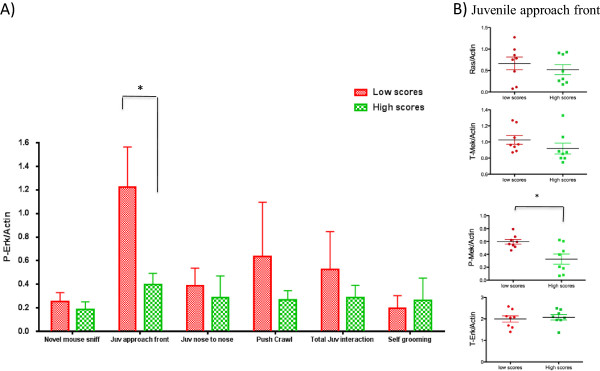
**Association between the degree of** ***MAPK*** **/ERK activity in the prefrontal cortex and social behavioral score was observed in F2 mice.** Western blot analysis of extracted proteins from the prefrontal cortex of eight mice with the lowest scores and eight mice with the highest scores of five different social behaviors were using the p-ERK antibody. **A)** Quantitative fold-changes of bands were assessed, after being normalized by actin levels. Mean p-ERK levels were increased significantly in F2 mice that performed poorly in the ‘juvenile approach front’ social assay using the non-parametric Mann-Whitney U test. **B)** Other *Mapk*/Erk signaling pathway proteins including Ras, t-Mek, p-Mek, and t-Erk were compared in mice with low scores on ‘juvenile approach front’ social assay versus mice with high scores, using Western blot analysis. p-Mek levels were also significantly amplified in mice with low juvenile approach front scores. (*P <0.05, bars represent mean values, whiskers show standard deviations). (Erk, Extracellular signal-regulated kinase; p-Erk, phosphorylated extracellular signal-regulated kinase; *Mapk*, Mitogen-activated protein kinase; Mek, Mitogen-activated protein kinase kinase; p-Mek, phosphorylated Mitogen-activated protein kinase kinase).

We next sought to determine whether increased activity levels of *Mapk*/Erk in the brain of BTBR mice were associated with the abnormal social behaviors of these mice. We previously developed a cohort of F2 intercrossed mice (B6 crossed with BTBR) in attempt to map the genetics of abnormal social behavior in this strain [13]. The 410 F2 mice were scored based on the following behavioral parameters: total juvenile interaction, juvenile approach front, juvenile pushing and crawling, juvenile nose-to-nose interaction, adult novel mouse sniffing, and self-grooming. For each social behavior, we selected the eight mice with the lowest scores and the eight mice with the highest scores (Figure 1). The median of each social behavior score was significantly different between the two groups of mice. Expression levels of p-Erk/actin in the prefrontal cortex were compared by Western blotting. We found that the degree of Erk phosphorylation was 2.7 fold higher in F2 mice with low scores on the ‘approach front’ social assay in comparison to mice with high scores (*P* = 0.032) (Figure 5A). This ratio was still significantly different between the two groups of F2 mice when incorporating total Erk into the equation: ((p-Erk/actin)/(t-Erk/actin); 2.7 fold greater in F2 mice with low scores, *P* = 0.037).

A similarly significant difference was observed when comparing the amount of p-Mek/actin (0.8 fold-change, *P* = 0.028), the signaling kinase immediately upstream of Erk (Figure 5C). However, the ratio of p-Mek/Mek was not significantly different between the two groups (*P* = 0.19). As in the parental strains, there was no difference in the total steady state protein levels of Mek and Erk molecules between the two groups (t-Mek, *P* = 0.104; t-ERK, *P* = 0.878). (Western blot lanes of these experiments are shown in Additional file 1).

Mice with other impaired social behaviors showed a trend towards higher p-Erk levels, but these changes were not statistically significant (Figure 5A). Prior studies have suggested that, in addition to the prefrontal cortex, the cerebellum may contribute to the development of social behaviors [21]. We therefore evaluated p-Erk levels in the cerebellum of F2 mice. In contrast to the prefrontal cortex, no significant differences were detected in the expression levels of p-Erk in the cerebellum of mice with low scores in social behaviors in comparison to mice with high scores (Additional file 4: Figure S1).

### *MAPK*/ERK expression in the brain and lymphocytes

**Figure 6:**
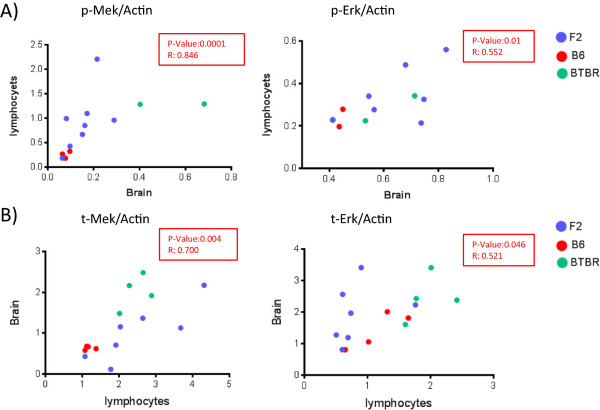
**Correlation between expression levels of** ***MAPK*** **/ERK in the brain and in lymphocytes.** Proteins were extracted from the brains and lymphocytes of four BTBR, four B6, and eight F2 mice. The expression and activity levels of MEK and ERK were compared between the brain and lymphocytes of each mouse, using Western blot analysis. **A)** The phosphorylation levels of MEK and ERK in the brain and lymphocytes of mice were significantly correlated using Spearman’s rank correlation coefficient analysis. **B)** Significant correlation in total expression levels of Mek and Erk proteins between the brains and lymphocytes of mice. BTBR: green dot, B6: red dot, F2: blue dot. (Erk, Extracellular signal-regulated kinase; p-Erk, phosphorylated extracellular signal-regulated kinase; *Mapk*, Mitogen-activated protein kinase; Mek, Mitogen-activated protein kinase kinase; p-Mek, phosphorylated Mitogen-activated protein kinase kinase).

The above findings suggest that p-Erk levels in BTBR mice are elevated and may correlate with the degree of social impairment. Given that other mouse models and human syndromes associated with ASD also show elevated activation of Erk, we sought to assess whether the elevated p-Erk that we observed in the cerebral cortex of BTBR mice was also seen in other tissue types, in particular lymphocytes, given their ready accessibility by venipuncture. We therefore isolated proteins from the brain and splenic lymphocytes of four BTBR, four B6, and eight newly bred F2 mice (from a cross of BTBR and B6), which should have a mixture of BTBR and B6 gene alleles and thus phenotypically should span the levels seen in the parental strains. Expression and activity levels of Mek and Erk were evaluated in these mice, using Western blot analysis, and normalized by actin levels. This analysis demonstrated a positive correlation between the amounts of p-Mek as well as p-Erk levels in the brain versus lymphocytes (p-Mek: Spearman’s rank correlation coefficient (r) = 0.846 *P* <0.001; p-ERK: r = 0.552, *P* = 0.017). Total expression levels of Mek and Erk were also significantly correlated between brain tissue and lymphocytes (t-Mek: n = 16, r = 0.700, *P* = 0.004; t-ERK: n = 16, r = 0.521, *P* = 0.046) (Figure 6).

## Discussion

In this study, we observed increased activation of the Ras/Erk pathway in the brains of BTBR mice, a strain that has social and behavioral deficits that may have relevance to ASDs [13]. Increased p-Erk levels in the newborn BTBR strain are consistent with a prior study that evaluated this pathway in BTBR mice [14]. However, this increased activation of Erk was less pronounced in adolescent BTBR mice. We also showed that total protein levels of Erk in the brains of BTBR mice are not increased compared to B6 mice. Thus, we hypothesize that the possible contribution of the *Mapk*/Erk signaling pathway to neurocognitive impairment in this mouse model of autism occurs through posttranslational modification of the Erk pathway. In support of this hypothesis, we showed that activation of the Erk signaling pathway, but not total Erk protein levels in prefrontal cortex, is associated with the degree of social impairment in intercrossed F2 mice with a mixed genetic background.

How might a change in the levels of ERK activation lead to behavioral abnormalities? Increased activation levels of *MAPK*/ERK in neurons have been shown to selectively increase pools of mRNAs encoding adhesion molecules and scaffolding proteins [22, 23]. Increased expression levels of these molecules can change the balance between excitatory and inhibitory synapses [23], which has been suggested as a likely basis for impaired cognition and possibly for ASD [24, 25]. In support of these hypotheses, Seese *et al*. showed a negative correlation between the intensity of synaptic p-Erk1/2 immunolabeling and cognitive function across BTBR mice [26]. This is clearly a complex issue, as the RAS/ERK signaling pathway is embedded in a network of other signaling pathways. ERK1/2 also has more than 70 different cytoplasmic and nuclear substrates, regulating many fundamental cellular processes. It is possible that each of the different proteins in this network has a cumulative effect on cognitive and behavioral impairments. However, more focused experiments are required to evaluate the role of each kinase individually. In contrast to the prefrontal cortex, when we evaluated the cerebellum of F2 mice, p-Erk levels were similar between mice with high and low social behavior scores, suggesting that the cerebellum may not be a focus of behavioral changes in this mouse strain. However, because we were only able to evaluate a limited number of F2 mice, we cannot rule out alpha or beta errors in our preliminary findings.

Because the *MAPK*/ERK pathway activation has been shown in many cell types to be central for cell division and differentiation, we evaluated whether BTBR mice had, in addition to elevated p-Erk, changes in cell proliferation and apoptosis in the developing forebrain. Evaluation of these parameters during a critical window of cerebral cortex development revealed distinct spatio-temporal changes in proliferation, but not apoptosis. Decreased cellular proliferation in the cerebral cortex throughout this developmental period is consistent with other studies that reported a robust reduction in neurogenesis in adult BTBR mice [27], which also lead to decreased brain volume and cerebral white matter in this strain [28]. To explore the role of *Mapk*/Erk on neural cell proliferation, Yang *et al*. overexpressed c-Raf in cultured cortical neurons. They observed impairment in neuronal cell differentiation and maturation and unchanged apoptosis in the presence of amplified *Mapk*/Erk, which is consistent with our findings [19]. However, the role of the *Mapk*/Erk pathway in regulating neuronal cell division and maturation is complex, as other studies have demonstrated that decreased *Mapk*/Erk activation can also disrupt progenitor proliferation [29–31]. Indeed, it is possible that any change from a homeostatic balance in this pathway may alter cognition and behavior. However, we were unable in this study to directly compare proliferation with behavior, so it is still remains possible that this change represents strain-to-strain variation without correlation with behavior.

In addition to these findings in the developing brain, we also found that the degree of activation of the Erk pathway directly correlated when comparing brain tissue to lymphocytes, suggesting that biological correlates of social impairment could be measured in peripheral blood. Based on these findings in a mouse strain, we speculate that this pathway could be dysregulated in the lymphocytes of autistic patients. Further investigation will be required to assay the levels of *MAPK*/ERK in the lymphocytes of individuals with autism and whether this could serve as a framework for developing a potential biomarker for autism.

## Conclusion

Our observations in BTBR mice as a preclinical model of autism, suggest that dysregulation of the ERK signaling pathway in CNS may be an important mediator of social behavior particularly in prefrontal cortex, In addition, measuring activation of this pathway in peripheral lymphocytes may serve as a surrogate marker for CNS ERK activity, and possibly autistic behavior.

## Electronic supplementary material

Additional file 1: Figure S4: Evaluating the expression levels of proteins with Western blot analysis. A) Western blot lanes of six BTBR versus six C57BL/6 mice. Brain lysates were evaluated at P0 and P30 time points with antibodies against RAS, p-ERK and actin. B) Western blot lanes of extracted proteins from the prefrontal cortex of eight F2 with the lowest scores and eight mice with the highest scores in the ‘juvenile approach front’. Brain lysates were evaluated with antibodies against RAS, t-MEK, p-MEK and p-ERK. C) Western blot lanes of extracted proteins from the prefrontal cortex of eight mice with the lowest scores and eight mice with the highest scores in ‘Juvenile nose-to-nose’, ‘Juvenile mouse-to-mouse sniff’ and ‘Total juvenile interaction’ social behaviors, using the p-ERK and actin antibodies. (PDF 177 KB)

Additional file 2: Figure S2: Quantification of cellular proliferation at E14 and E17 indicate that BTBR mice have altered neurogenesis. E14 and E17 control (n = 6 per age) and BTBR mice (n ≥6 per age) were injected with EdU 30 minutes prior to sacrifice and labelled for either PH3, Pax6, or Tbr2 in combination with Ki67 and EdU. Cell counts from representative regions of the cortical midline (A-B), neocortex (C-D), and ganglionic eminence (E-F) were performed for single, double, and triple-labelled cells. Data are represented as mean ± SEM. Mann-Whitney U test for significance: *P <0.05, **P <0.01, ***P <0.001. (PDF 345 KB)

Additional file 3: Figure S3: The ganglionic eminence of BTBR and control mice expresses low levels of the progenitor markers Tbr2 and Pax6. E14 control (A), E14 BTBR (B) mice, E17 control (C), and E17 BTBR mice (D) were injected with EdU 30 minutes prior to sacrifice and immunolabelled for nuclear marker DAPI (white or blue), EdU (green), and either Pax6 or Tbr2 (red). High power images (A’-D”) demonstrate that neither Tbr2 nor Pax6 are expressed at detectable levels in the ganglionic eminence. Scale bar in B and D represents 500 μm for A, B and C, D respectively. Scale bar in D” represents 100 μm for A’-D”. n ≥6 for all conditions. (PDF 4 MB)

Additional file 4: Figure S1: The association between degree of *Mapk*/Erk signaling pathway activity in the cerebellum and social behavior scores in F2 mice. A) Proteins were isolated from the cerebellum of eight F2 mice on the extremities of each social behavior. p-Erk levels were evaluated in two groups of mice with lowest and highest scores of each social behavior, using Western Blot analysis. B) Quantitative fold-change in p-Erk have been shown for each social behaviors, after being normalized by actin. No significant change in p-Erk levels were detected in comparing mice with low social behavioral scores and mice with high scores. (PDF 200 KB)

## Abbreviations

ASD: Autism spectrum disorders
B6: C57BL/6 J
BTBR: BTBR T + tf/J
CNS: Central nervous system, CNV, Copy number variant
DAPI: diamidino-2-phenylindole
ERK: Extracellular signal-regulated kinase
GE: Ganglionic eminence
HRP: horseradish peroxidase
LTD: Long-term depression
LTP: Long-term potentiation
*MAPK*: Mitogen-activated protein kinase
MEK: Mitogen-activated protein kinase kinase
Pax6: Paired Box 6
PBS: Phosphate buffered saline
p-ERK: Phosphorylated extracellular signal-regulated kinase
PH3: Phospho-histone H3
RBC: Red blood cell
t-ERK: Total protein level of ERK
Tbr2: T-Box Brain Protein 2.
