# Supplementary figures and images for: Mapk/Erk activation in an animal model of social deficits shows a possible link to autism
Source: Mol Autism. 2014 Dec 22;5:57. doi: 10.1186/2040-2392-5-57 (PMC4396809; doi:10.1186/2040-2392-5-57)

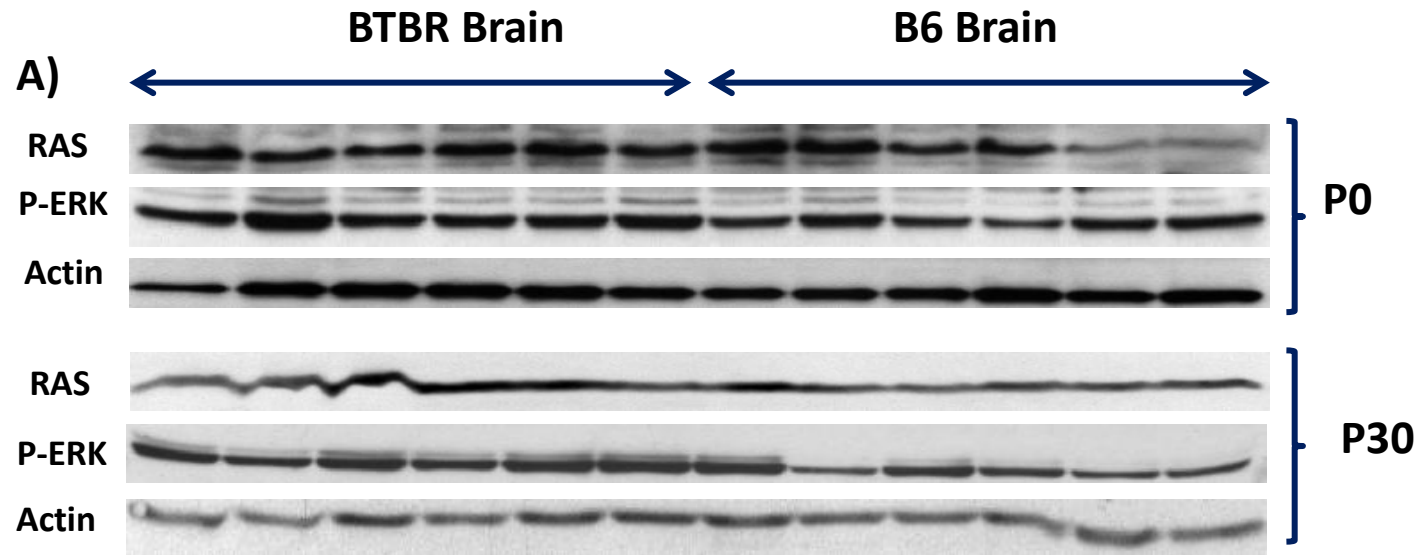

**B)**

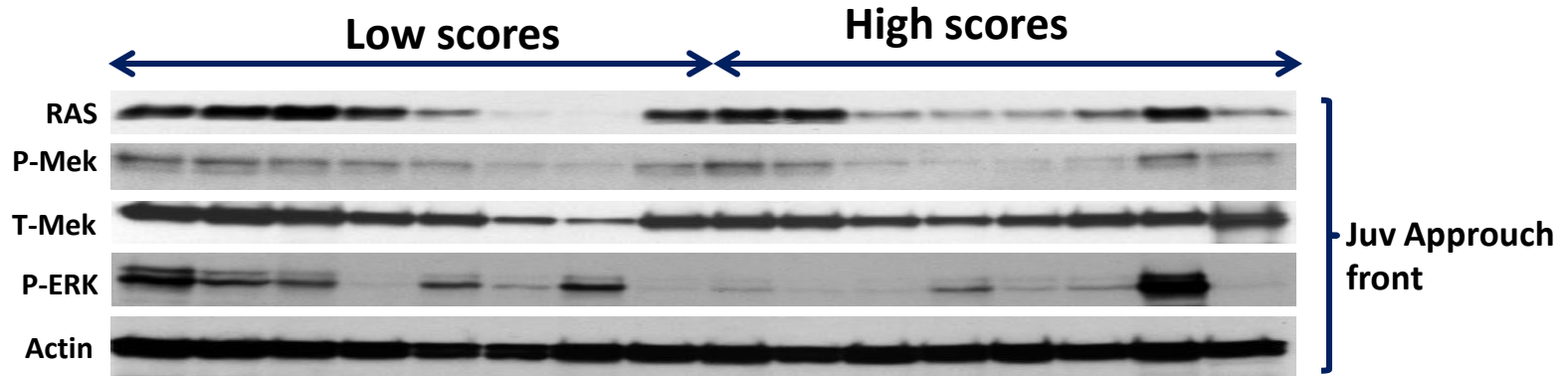

**C)**

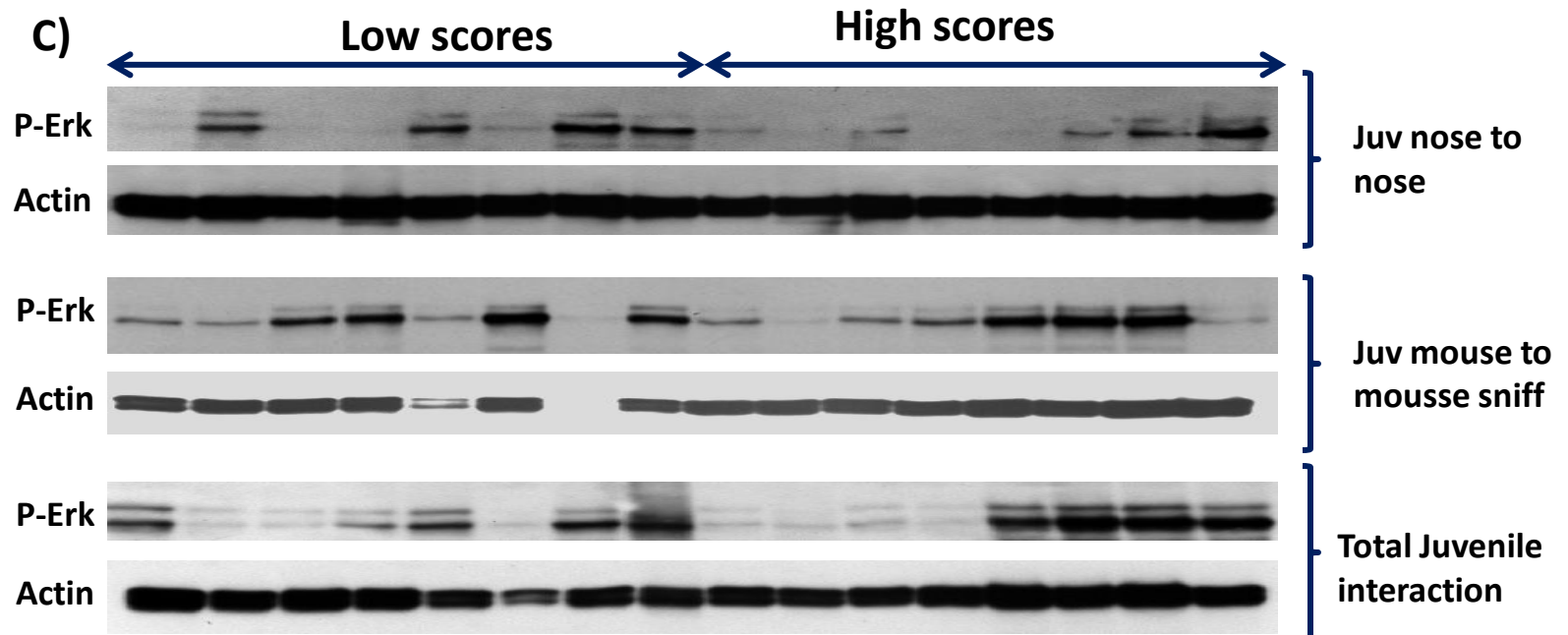

Supplement: Supplementary file 1 — Additional file 1: Figure S4: Evaluating the expression levels of proteins with Western blot analysis. A) Western blot lanes of six BTBR versus six C57BL/6 mice. Brain lysates were evaluated at P0 and P30 time points with antibodies against RAS, p-ERK and actin. B) Western blot lanes of extracted proteins from the prefrontal cortex of eight F2 with the lowest scores and eight mice with the highest scores in the ‘juvenile approach front’. Brain lysates were evaluated with antibodies against RAS, t-MEK, p-MEK and p-ERK. C) Western blot lanes of extracted proteins from the prefrontal cortex of eight mice with the lowest scores and eight mice with the highest scores in ‘Juvenile nose-to-nose’, ‘Juvenile mouse-to-mouse sniff’ and ‘Total juvenile interaction’ social behaviors, using the p-ERK and actin antibodies. (PDF 177 KB) [file 13229_2014_148_MOESM1_ESM.pdf]

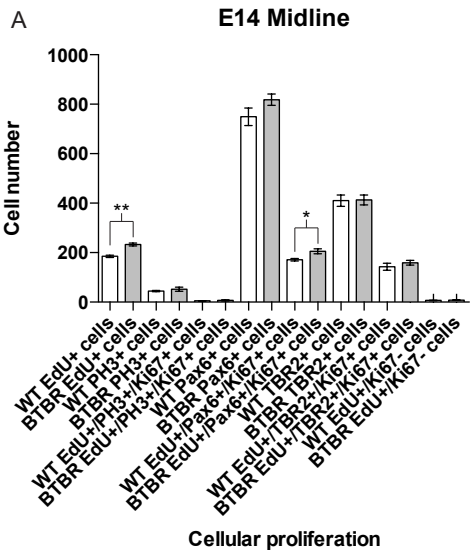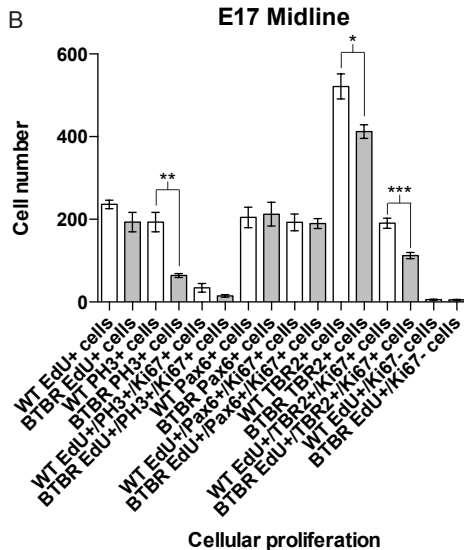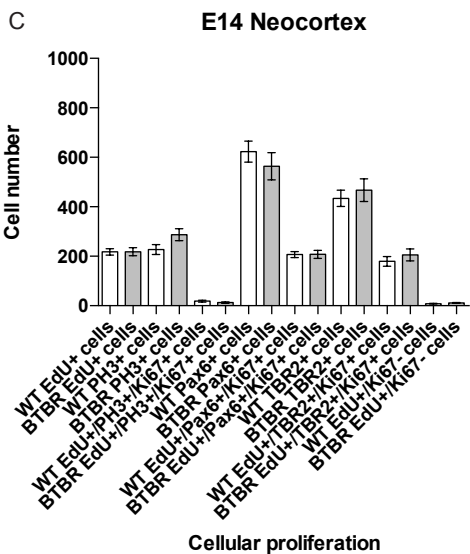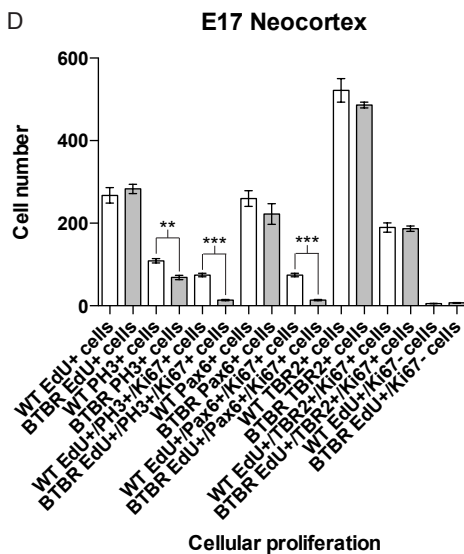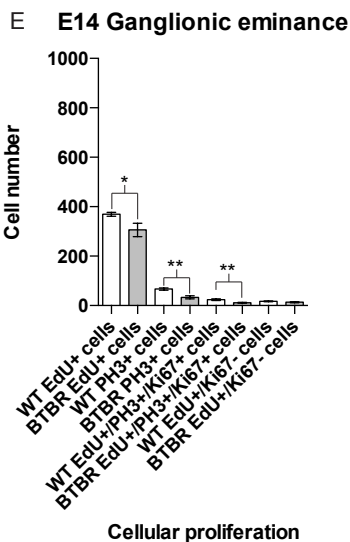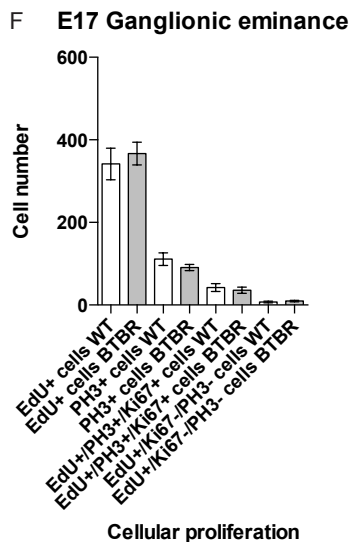

Supplement: Supplementary file 2 — Additional file 2: Figure S2: Quantification of cellular proliferation at E14 and E17 indicate that BTBR mice have altered neurogenesis. E14 and E17 control (n = 6 per age) and BTBR mice (n ≥6 per age) were injected with EdU 30 minutes prior to sacrifice and labelled for either PH3, Pax6, or Tbr2 in combination with Ki67 and EdU. Cell counts from representative regions of the cortical midline (A-B), neocortex (C-D), and ganglionic eminence (E-F) were performed for single, double, and triple-labelled cells. Data are represented as mean ± SEM. Mann-Whitney U test for significance: *P <0.05, **P <0.01, ***P <0.001. (PDF 345 KB) [file 13229_2014_148_MOESM2_ESM.pdf]

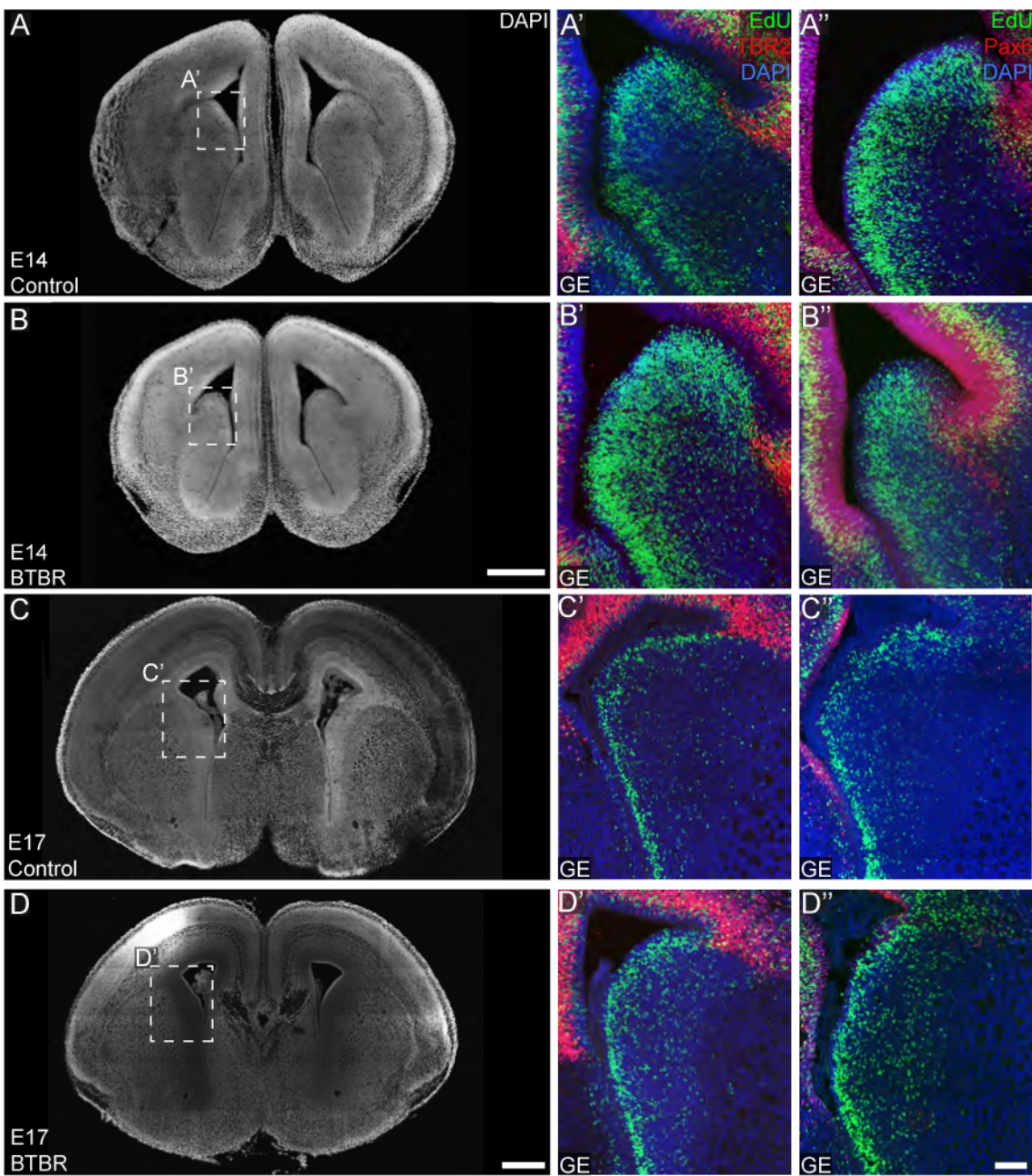

Supplement: Supplementary file 3 — Additional file 3: Figure S3: The ganglionic eminence of BTBR and control mice expresses low levels of the progenitor markers Tbr2 and Pax6. E14 control (A), E14 BTBR (B) mice, E17 control (C), and E17 BTBR mice (D) were injected with EdU 30 minutes prior to sacrifice and immunolabelled for nuclear marker DAPI (white or blue), EdU (green), and either Pax6 or Tbr2 (red). High power images (A’-D”) demonstrate that neither Tbr2 nor Pax6 are expressed at detectable levels in the ganglionic eminence. Scale bar in B and D represents 500 μm for A, B and C, D respectively. Scale bar in D” represents 100 μm for A’-D”. n ≥6 for all conditions. (PDF 4 MB) [file 13229_2014_148_MOESM3_ESM.pdf]

A)

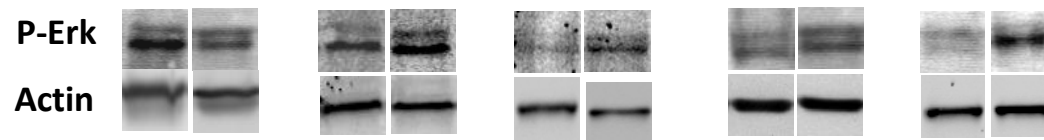

B)

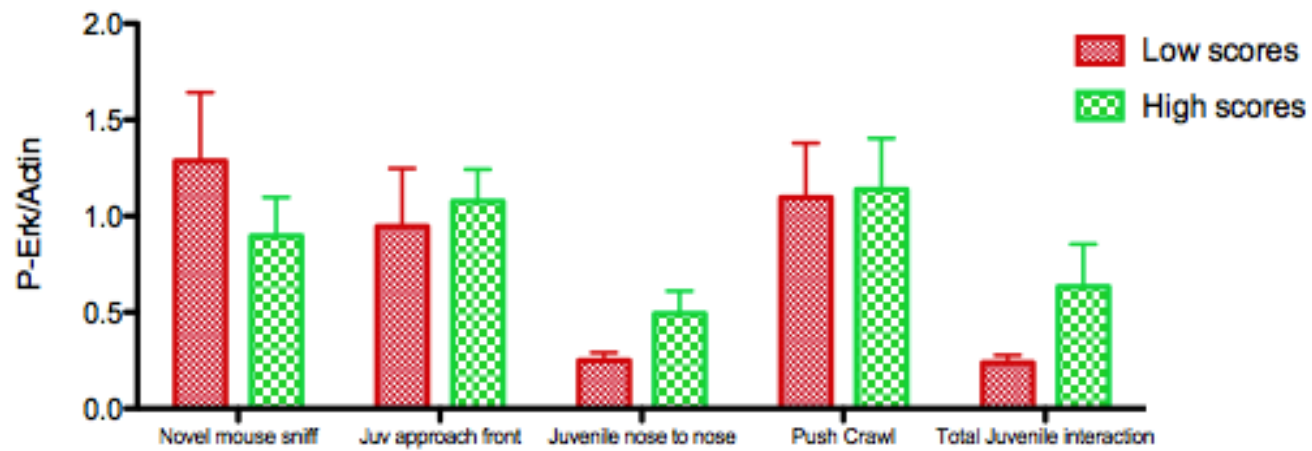

Supplement: Supplementary file 4 — Additional file 4: Figure S1: The association between degree of Mapk/Erk signaling pathway activity in the cerebellum and social behavior scores in F2 mice. A) Proteins were isolated from the cerebellum of eight F2 mice on the extremities of each social behavior. p-Erk levels were evaluated in two groups of mice with lowest and highest scores of each social behavior, using Western Blot analysis. B) Quantitative fold-change in p-Erk have been shown for each social behaviors, after being normalized by actin. No significant change in p-Erk levels were detected in comparing mice with low social behavioral scores and mice with high scores. (PDF 200 KB) [file 13229_2014_148_MOESM4_ESM.pdf]
